# Food Marketing as a Special Ingredient in Consumer Choices: The Main Insights from Existing Literature

**DOI:** 10.3390/foods9111651

**Published:** 2020-11-12

**Authors:** Vítor João Pereira Domingues Martinho

**Affiliations:** Agricultural School (ESAV) and CERNAS-IPV Research Centre, Polytechnic Institute of Viseu (IPV), 3504-510 Viseu, Portugal; vdmartinho@esav.ipv.pt

**Keywords:** literature survey, Scopus, brands, consumer preferences

## Abstract

The choices and preferences of food consumers are influenced by several factors, from those related to the socioeconomic, cultural, and health dimensions to marketing strategies. In fact, marketing is a determinant ingredient in the choices related to food consumption. Nonetheless, for an effective implementation of any marketing approach, the brands play a crucial role. Creating new brands in the food sector is not always easy, considering the relevant amount of these goods produced within the agricultural sector and in small food industries. The small dimension of the production units in these sectors hinders both brand creation and respective branding. In this context, it would seem important to analyse the relationships between food marketing and consumer choice, highlighting the role of brands in these frameworks. For this purpose, a literature review was carried out considering 147 documents from Scopus database for the topics of search “food marketing” and “choices” (search performed on 16 October 2020). As main insights, it is worth highlighting that the main issues addressed by the literature, concerning food marketing and consumer choices, are the following: economic theory; label and packaging; marketing strategies; agriculture and food industry; market segments; social dimensions; brand and branding. In turn, food marketing heavily conditions consumer choices; however, these related instruments are better manipulated by larger companies. In addition, this review highlights that bigger companies have dominant positions in these markets which are not always beneficial to the consumers’ objectives.

## 1. Introduction

The food choices by consumers are influenced by several factors, where the prices traditionally have great importance, as highlighted by the economic theory. However, there are new tendencies, and some segments currently privilege healthy [1] and sustainable characteristics [2]. Food consumption has several dimensions, including that of a social and cultural magnitude, and this sometimes compromises policies to change unadjusted behaviours [3] and influence food perceptions [4]. The sociodemographic and behavioural factors also have their implications [5] on consumer behaviour. On the other hand, labelling and packaging have a significant impact on consumer choices and preferences [6].

In these contexts, marketing strategies are useful and powerful approaches in order to create and maintain a market in any economic sector and, specifically, in the food industry [7]. However, in the food market, it is important to distinguish two production sectors, agriculture and industry. These two distinct sectors with different dynamics have implications on the respective markets. This is important to highlight, because this makes the food sector different from other economic sectors.

Agriculture has several particularities that constrain the design of effective marketing plans. In fact, the structural context of farms, often, in small dimensions, in great numbers and the producing commodities are limited in the ability to create a custom positioning, a crucial ingredient for any marketing approach. The main problem of this atomised structure is associated with the reduced individual level of production, focused on parts of the year that prove difficult to maintain a regular presence in the market and the respective branding. These weaknesses of the sector limit the market choices of farmers [8]. Of course, the brand and the agricultural sector are only a part of the food marketing framework.

In turn, the food industry is often conditioned to be more competitive and to generate value added through the creation of brands. In fact, this is a sector with the dynamics and the competitiveness predicted by the economic theory for the industry, i.e., as having activities with increasing returns to scale. The performance in terms of productivity and efficiency allows for another presence in the markets and possibilities to further develop marketing plans and strategies for a more sustainable development [9].

Considering that marketing approaches influence consumer food choices, the literature survey highlights the relevance of a systematic review concerning two dimensions: food marketing and consumer choices, taking into account the specificities of the two sectors related to food production.

From this perspective, the research carried out intends to highlight the main insights from the scientific literature into the relationships between food marketing and the choices of consumption performed by consumers. To achieve this objective, 147 documents (only articles and reviews) from the Scopus database [10] were obtained, considering as topics for searches carried out on 16 October 2020 “food marketing” and “choices”. These documents were analysed through a literature survey. To better perform the literature analysis, a previous bibliographic analysis and literature survey were considered, and this approach allowed for organisation of the literature review with the following structure: economic theory; label and packaging; marketing strategies; agriculture and food industry; market segments; social dimensions; brand and branding. This approach was complemented using the PRISMA (Preferred Reporting Items for Systematic Reviews and Meta-Analyses) methodology [11]. For the PRISMA approach, 137 documents (only articles and reviews) were also considered from the Web of Science Core Collection [12] for the same topics. When the documents from Scopus and Web of Science were considered together, through the Zotero software [13], a great majority were duplicated (around 100). From this perspective, considering the relevant number of documents duplicated across the two scientific databases and the Scopus platform having more documents, the decision was made to opt only for the documents from this database. The topics of search “food marketing” and “choices” were selected to find documents in the scientific databases related to the interrelationships between food marketing and consumer choices. The search topics “food”, “marketing”, and “choices” could be considered, for instance, but this search option would greatly increase the number of documents found, taking the level to an infeasible amount for a literature review; furthermore, the studies obtained were outside the intended scope (“food marketing”).

## 2. Bibliographic Sample Characterisation

The information presented in this section is relative to a sample obtained from the Scopus database for a search carried out with the following topics/keywords: “food marketing” and “choices”. In addition, it is important to highlight that the identification of the sample and its analysis considered other scientific contributions concerning systematic reviews [14,15,16,17].

**Figure 1:**
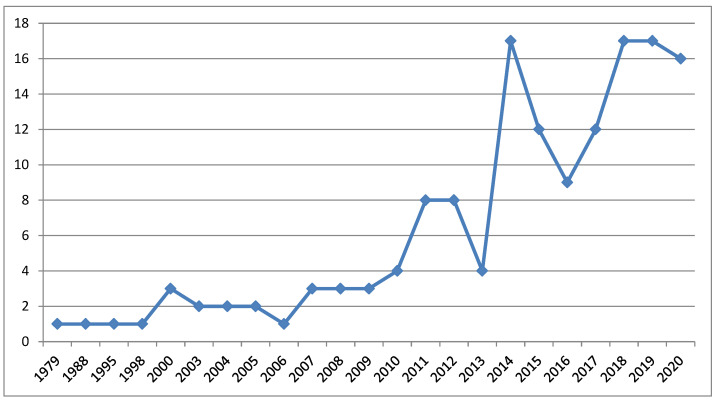
Distribution of the documents across years.

The number of documents related to the topics considered has increased from 1970 until today, with relevant breaks in 2013 and 2016, with a total of 16 documents in 2020 (Figure 1). This context shows that there are opportunities to increase the number of documents published with regard to these fields, considering the annual average number of studies published and the relevance of the topics.

**Figure 2:**
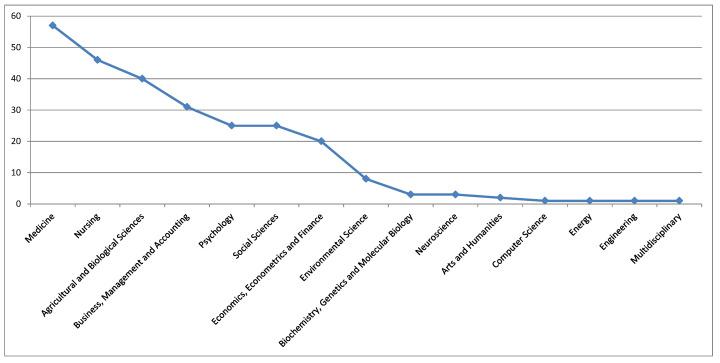
Distribution of the documents across subject areas.

A large part of the documents focused on subject areas such as the following (Figure 2): medicine; nursing; agricultural and biological sciences; business, management and accounting; psychology; social sciences; economic, econometrics, and finance; and environmental science. This framework reveals the multidisciplinary dimension of the issues related with the topics addressed here.

**Figure 3:**
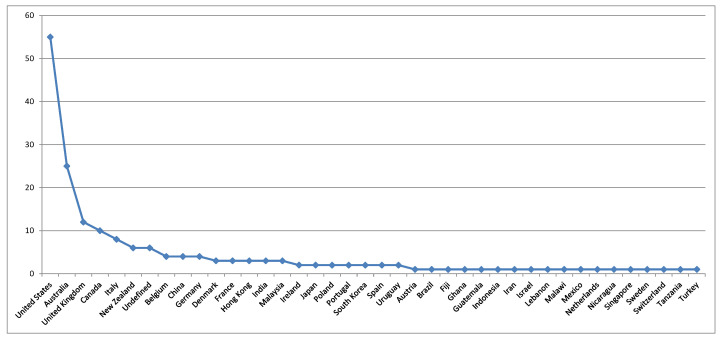
Distribution of the documents across countries.

The majority of the studies were carried out by authors affiliated to institutions from the United States, Australia, the United Kingdom, Canada, Italy, New Zealand, Belgium, China, and Germany (Figure 3). The several dimensions associated with these topics are relevant to several countries around the world. In this way and considering the values presented in Figure 3, there are opportunities to be further explored regarding these topics by affiliated authors in institutions from important countries, such as, China, India, Brazil, and the European Union member-states. 

**Figure 4:**
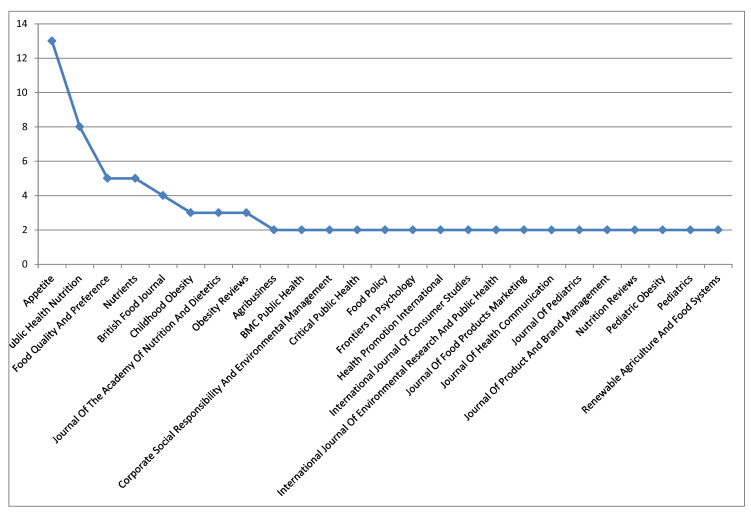
Source titles with two or more documents.

Source titles having two or more documents are those presented in Figure 4. The following journals were noted: Appetite (13); Public Health Nutrition (8); Food Quality and Preference (5); Nutrients (5); British Food Journal (4); Childhood Obesity (3); Journal of the Academy of Nutrition and Dietetics (3); Obesity Reviews (3).

**Figure 5:**
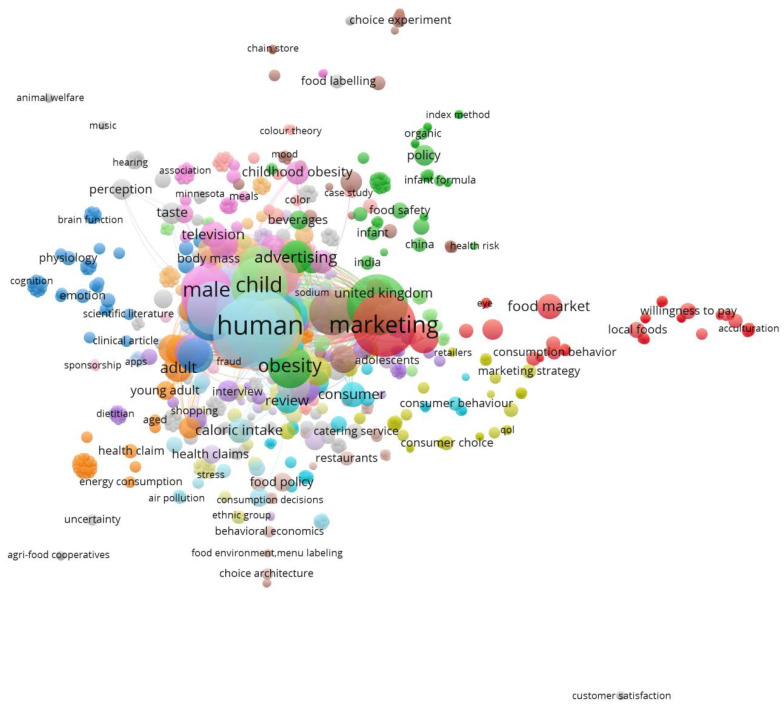
Co-occurrences of all keywords (one as a minimum number of occurrences of a keyword).

Figure 5 was obtained using VOSviewer software [18,19] with the 147 documents obtained from the Scopus database. This figure was obtained using bibliographic data for co-occurrence links and all keyword items. In this figure, the circle/label size represents the number of keyword occurrences, and relatedness (proximity of circles/labels) is determined on the basis of the number of documents in which the keywords occur together [19]. Figure 5 highlights the relevance of keywords, for example, obesity, child, advertising, review, interview, adolescents, market, policy, labelling, perception, willingness to pay, health, choice experiment, index method, case study, apps, and television. These keywords reveal some relevant dimensions related to food and marketing and consumer choices (obesity, health, children and youths, labelling, perceptions, taste, willingness to pay, policies, and media) and some methodological approaches (review, interview, choice experiment, index method, and case study). On the other hand, there is a great amount of relatedness (number of documents in which the keywords occur together) between food marketing and human obesity, especially in men and children. 

## 3. Literature Survey

Considering the bibliographic analysis and a preliminary literature survey, this section is divided into the following subsections: economic theory; labelling and packaging; marketing strategies; agriculture and food industry; market segments; social dimensions; brand and branding.

### 3.1. Economic Theory

As predicted by the theory of demand, the consumption of goods and services by consumers to satisfy their daily needs is dependent on market prices. In addition, the theory of utility explains that, when consumers intend to satisfy their needs, they also expect to maximise utility, depending on their income. This is true in every market, including in the food markets from low-income countries [20]. Consumer demand is dependent on several factors, but the prices (own product, substitute product, and complementary product prices) are amongst the most important variables. Of course, other variables, such as product quality and the economic conjuncture of each country, have their influences on consumption. In these frameworks, consumers combine quantities of goods and services so as to obtain the maximum satisfaction from their consumption. The level of satisfaction achieved is dependent on the available revenue to consume. The economic theory assumes that the economic agents are rational, and this means that consumers want to consume more when prices are lower with the exception of luxury products or goods and services of basic needs [21]. The marketing plans, in general, bear these contexts in mind, because the consideration of these fields is determinant for a successful strategy in the food sector.

On the other hand, some dimensions are multidisciplinary and networked, such as those, for example, related to welfare [22]. Welfare is, in fact, the focus of research for several disciplines such as biology, economy, psychology, and sociology. This transversal perspective could prove interesting as a means for cross-approaches, including insights from economic theory, to promote more adjusted patterns of food consumption, mainly those more compatible with health requirements [23]. The impact on health from food consumption is a concern for several stakeholders; however, it is not an easy challenge to mitigate these implications, due to the market power of certain stronger brands.

Economic options and the respective economic dynamics, with consequences on prices and on consumer incomes, have direct and indirect impacts on food choices and, consequently, on the health of the respective population [24]. In turn, the economic theory may provide interesting insights for more effective health policies and programmes that incentivise, in a greater way, food choices which are more compatible with a balanced human life environment [25]. The economic theory may also be a relevant ally towards supporting better knowledge about company frameworks for a more effective market and marketing approaches [26].

The price elasticities, for example, may provide relevant support in these strategies and enable us to predict future patterns of food consumption [27]. The prices do indeed have a determinant impact on food markets [28], despite their particular price and income elasticities. In general, the food markets, specifically, those more linked with the production sector (agriculture), have lower, inelastic price elasticities. This means that the consumers are not sensitive in their consumption to price changes, mainly due to the fact that food products are often essential goods and services of basic needs and where the prices are lower. The same happens for income elasticities, meaning that, when consumers have more revenue, they have a tendency to increase their consumption of products other than food goods. In other words, when consumer income increases, they are willing to increase industrial and service consumption rather than consume more food [21]. This is a great task for the food industry, where the brand and respective branding are called upon here to play their contribution, whilst sometimes having implications on consumer health.

### 3.2. Label and Packaging

Food labelling and packaging are used to inform consumers about the product’s characteristics, in accordance with legislation, and for marketing purposes [29], but they may also provide support for healthier choices [30]. The legislation regulates the information which may be considered for labelling, and this can sometimes be too bureaucratic and may bring about additional difficulties to market strategies. For example, in some food/beverage sectors, prior to any change in the label, there needs to be previous approval from the competent institutions, and this limits the strategic tasks of the respective companies, mainly when the intention is to provide something more personalised for the consumers.

Despite this regulation, the objectives of labelling to protect human health are, sometimes, compromised. The labelling text and design condition the perceptions of the consumers about food goods and services and influence their choices [31], especially when questions related to health are implicit [32]. The influence of the label design also has relevance in the perceptions and choices among children [33], where cartoon characters and nutritional statements have their importance [34].

The regional and Mediterranean labels are, in general, designed to promote marketing strategies and highlight product attributes [35]. The regional brands and respective labels are ways to highlight local food characteristics and to create value added in endogenous resources. In fact, the big challenge in some food sectors is to create value added for stakeholders, and these regional brands support the objectives to bring more value added to several operators. In general, these regional brands are umbrella products that promote other endogenous goods and services. 

The type of packaging has an influence on consumer perceptions about the healthfulness of the respective food. For example, milk in glass packaging is perceived as being healthier than milk packaged in a carton [36]. Packaging influences children and adults in different ways. For example, for adults, the package size and shape are important attributes, more than the information present on the labels [37]. Different generations have distinct patterns of consumption, and millennials, having a different educational environment, where social media has a great impact, have other preferences and vulnerabilities.

Nonetheless, the labelling and packaging are, in some cases, more useful in aiding consumers to identify healthier food rather than trying to influence them to buy these products [38]. In addition, the presence of cartoons on packages positively influences children to choose fruit and vegetables, but this is unfortunately used more for choices of energy-dense and poor nutritional foods [39]. Cartoon characters on packaging do in fact have a great impact on children’s food choices [40]. The taste perceptions are determinant for children’s choices, and the packaging design influences these assessments. Children identify the product name, prices, and images as being the most relevant packaging characteristics for their choices [41]. The information that stimulates human sensations, such as images and songs, is powerful in influencing consumers.

Sometimes, some information on the packaging may mislead consumers about the real properties of the food chosen [42] or does not conveniently inform consumers about the nutritional characteristics [43]. This is particularly disturbing in some nutritional and health claims [44]. The messages on the packaging must be clear [45] and appropriate for what the products really are [46]. 

In general, researchers seem to agree on the need for some control by legislation of the information present on packaging [47], primarily that which promotes unhealthy food choices [48] in children [49]. These concerns are transversal around the world, including, for example, studies carried out in Brazil [50], Australia [51,52,53], United States (US) [54,55], since the 1970s [56], India [57,58], Philippines [59], Malaysia [60], and Ireland [61]. In any case, the decisions related to regulation towards preventing health issues should bear in mind the international commitments and consequent constraints [62].

From another perspective, health standards are sometimes not uniform across organizations and countries [63]. This may create additional difficulties for the producers and retailers who operate in international markets. It could be important, for example, in the context of the World Trade Organization or the World Health Organization, to find transversal standards for the domains relative to healthy food attributes. 

### 3.3. Marketing Strategies

Food marketing is an important tool [64] to build and maintain markets through the creation of ties of confidence and loyalty between the producers/sellers and the consumers. Food marketing is dependent on several different dimensions, especially those related to the particularities of the sectors associated with food goods and services; in this way, the marketing plans are no easy task [65].

In any circumstance, the marketing of food as an external factor which influences consumer choices [66] is a powerful instrument that may be used to promote public campaigns, such as those related to healthy eating [67] across the several points of food sale, including restaurant kids’ menus [68] and supermarkets [69]. However, for companies, the trade-off between health and profit is not easy to solve and this is visible in many of the strategies adopted.

For example, supermarket checkout areas are especially strategic for marketing plans and deserve special attention in terms of their impact upon human health [70]. From another perspective, the tie-in offers in fast food menus for children could be restricted to healthy promotions [71]. The same concern could be present when sport celebrities are associated with the marketing plans [72] for children and parents [73] or in the criteria used to choose sport sponsors [74]. In turn, in the definition of marketing approaches, the message for healthy food promotions should be clear, well designed, and well oriented [75] to avoid misunderstandings [76], principally by children [77], as well as to obtain the intended objectives [78].

The media is a determinant way to communicate with consumers [79], which calls for adjusted advertising when it comes to promoting healthy consumption. However, often times, the consumers, especially youths, are not prepared to deal with these aggressive forms of publicity [80] and are not able to decide on the most important information [81], explicitly that which is related to nutritional characteristics [82]. In fact, the youth and children who are more engaged with, for example, social media are more vulnerable to being influenced into buying unhealthy food [83].

The marketing strategies designed by food operators are very persuasive, and this implies that the consumers who are exposed to food marketing campaigns seem to be more prone to agreeing with their strategies, including those for unhealthy food choices [84]. The television and internet seem to be the most powerful ways to influence exposed consumers [85], specifically through neuromarketing approaches which encourage children to favour taste when making food choices [86]. Television cooking shows are particularly influential on the consumption patterns of children and the youth [87]. The same happens on children’s websites [88] and social media [89]. The taste is, indeed, a decisive ingredient in food marketing strategies [90] and, usually, food marketing uses contexts related to this attribute to design its plans and influence customers.

Neuromarketing is an emergent technique that applies approaches to measure spontaneous reactions [91], with relevant impacts on the consumers’ choices [92], especially on young people [93]. The songs, image sequence, and colour are tools usually considered to support neuromarketing policies [94]. The evolution of these approaches allows for current expressions such as “musical flavour” to be normal and accepted by the several stakeholders [95]. Usually, consumers are influenced in their consumption without any perception of this factor. The stimuli for human senses have a strong impact on the consumers’ perceptions, and these tools are used to intentionally encourage consumers by marketing professionals in a subconscious way.

Magazines, as well as television and the internet, are powerful ways to advertise to consumers [96], sometimes in a more persuasive way [97]. This is because, in some cases, the control approaches are more focused on television and the internet, whilst the written forms of advertisement are forgotten about although they do have similar tools to influence consumers.

The several strategies related to food marketing have an impact on dietary choices, consumption preferences, and cultural values [98]. These changes in the pattern of consumption, as a consequence of food marketing, are particularly visible in countries that became more vulnerable to external advertisements, due to political, social, or a conjuncture of changes. In any event, a familiar environment and parents’ behaviour have a determinant impact on the several food choices [99].

An emerging area in the marketing of food is the guilt-free approach [100]; however, this a multidisciplinary field where several disciplines are called upon to add their contributions. It is important to find food marketing strategies that combine the profit aims of the companies with the health of consumers [101].

### 3.4. Agriculture and Food Industry

The food industry is interlinked with the agricultural sector, making this sector and its marketing strategy dependent on the options made by the farmers [102], specifically, in terms of farming practices compatible with the environment and animal welfare [103], as well as with the safety of the products themselves [104]. For example, organic farming products may have for the food markets a set of virtues and advantages, relative to conventional agriculture, but may also bring about a set of barriers and difficulties (because of the higher prices, for example) [105]. In any case, farming practices which are compatible with the environment will be the future in many countries around the world, especially in the European Union member-states. In fact, the several measures of the Common Agricultural Policy (CAP), mainly since 1992, have gone in this very direction. Due to structural and environmental problems, the CAP since 1992 has become more directed towards promoting sustainable development in an integrated rural approach, where the agri-environmental (organic farming, integrated production, etc.) measures have gained more relevance. The recent instruments created in the CAP framework, such as Greening, are examples of an agricultural policy which is more concerned about the environment within the European context [106,107].

Nonetheless, the food industry is an interesting way to bring about value added to agriculture, because, in farms, due to their characteristics, marketing strategies have, in certain circumstances, less importance in the market than other factors [108]. Agriculture as a sector of food commodities has additional difficulties in order to be presented into the market in a differentiated way, and this compromises marketing strategies.

The Protected Designation of Origin (PDO) products and the associated producers’ organizations are examples that may support some market differentiation and provide more structured and effective marketing strategies [109]. These PDO and the respective certification brands allow for the protection of local and regional food attributes and are interesting tools to create marketing strategies common to the respective stakeholders. Of course, the PDO brands are not the same as individual trademarks, but may bring interesting contributions, primarily for smaller farmers, for example, with more budgetary difficulties to implement strategies complementary to production techniques, to create value added in the markets, and to increase their income.

The broad diversity of farms, in terms of size, characteristics, and organization, makes the agricultural sector specific, with particular dynamics that influence the strategies adopted for food marketing [110]. The different programmes and policies designed for the agricultural sector have relevant impacts on the agriculture industry’s dynamics [111] and implicitly on the respective markets [112]. This has been a concern for the several policymakers and policy design in the European Union context bearing in mind these agricultural market characteristics, but it continues to require some further adjustments for some local particularities.

Local markets appear, in general, as great opportunities for farmers who have achieved consumer preference or loyalty, principally in terms of quality [113]. These local markets are relevant ways to shorten the agricultural chain. In certain circumstances, consumers are willing to pay more for local food [114]. Usually, the greater margin of value added in agricultural markets remains with the intermediaries and the retailers. Local markets and short agri-food chains (farm events, farm tourism, farm shops, etc.) may support farmers to maintain a large part of the total amount of value added generated in the markets. Nonetheless, the channels used in the markets depend, in some cases, on their structural characteristics, mainly those linked with their experience in the sector [115].

In the agricultural food industry market, questions sometimes appear such as those related to patriotism, where dimensions associated with food safety may contribute to adjusted marketing strategies that provide support to overcome these aspects [116]. Consumers are concerned with the health impacts of food consumption and, in this way, are sensitive to claims associated with food safety.

For an effective marketing plan in the agricultural sector, considering their specificities, the associations and cooperatives are fundamental, when well managed and organised. However, sometimes, the management structure of these organizations is not the best adjusted, and this has consequences on the sector’s performance [117]. The associations and cooperatives are crucial for technical support to the farmers and to concentrate the agricultural supply of the farmers who have worse conditions and dimensions in terms of storing production. On the other hand, the output concentration allows further capacity to negotiate contracts and prices with retailers.

The new technologies of information and communication may be useful tools to support marketing strategies in farms, and some farmers are indeed willing to pay for electronic platforms [118]. Social media is one of the cheaper and easier ways to promote food products, and this may be used without relevant difficulties by the several stakeholders. Some years ago, publicity and advertising were expensive and restricted to the traditional means of communication, such as television, radio, newspapers, and magazines.

### 3.5. Market Segments 

Food markets are characterised by heterogeneous segments of consumers [119], involving a great diversity of realities [120], some more sensitized to health statements and others more influenced by nutritional information [121]. These contexts bring about interesting challenges for the marketing professional and for researchers, due to the great number of brands that operate in these markets. This diversity implies that food markets could be segmented considering food features, sales structure, and consumer characteristics [122].

Insufficient nutritional information seems to be one of the main factors that, in some segments, hinders the prevention of unhealthy food consumption [123]. This is particularly alarming in countries with a lower income [124]. Children and low-income consumers are vulnerable segments to persuasive and targeted marketing campaigns: children because of their lower skills to deal with marketing strategies to sell more and low-income consumers because of their vulnerability to lower-priced products.

As a result of these frameworks, the terms used to describe the nutritional dimensions, targeted at specific segments, need proper regulation, since the personal perceptions of consumers concerning the real definition of these expressions are not consensual [125] and this, therefore, opens up an element of free will for the marketing designers/strategists.

In some segments, the perceptions about food safety are more important for consumer choices than their socioeconomic characteristics [126]. In a similar pattern, consumers are, in some cases, prepared to pay more for beneficial health claims than for nutritional claims [127]. Nonetheless, the consumer’s choices of food with heath claims are, in general, interrelated with several factors, such as those related with the socioeconomic domains [128]. Depending on the segments considered, the food choices may be influenced by personality, health, sensory attributes, price, and convenience [129], as well as, by environmental, ethnic, and cultural contexts [130].

More adjusted regulations may support the promotion of more healthy advertising to more vulnerable segments [131]. However, there are areas that need to be worked on, across several segments, concerning regulations, recommendations, and policies. Some of these dimensions that deserve special attention are the accuracy [132] and the perception [133] of consumers relative to these fields associated with healthier food. The main fields to be considered by regulations to promote a healthier choice by children are the usual persuasive techniques such as promotional offers, nutrition and health claims, and appeals towards taste and fun [134].

Tourism is an important market segment that may bring significant contributions to food marketing strategies, considering the several interrelationships between the associated sectors in these interlinkages [135]. The relationships between food and tourism are well known and strong, and they should be considered in joint strategies to promote the two sectors in an integrated way. Nonetheless, the externalities that may be created in this common strategy could also spread positive effects to other sectors (transport, support services, etc.).

### 3.6. Social Dimensions

The interlinkages between the social responsibility of firms and the market response to the respective consumers are positive [136]; however, the traditional consumer determinants, such as the price, continue to be relevant [137]. The strong impacts from the level of prices on consumer choices are particularly problematic in lower-income countries and consumer segments [138]. Knowledge about price relevance in consumer choice may be further considered so as to promote heathy strategies and be complemented with nutritional education [139]. Adjusted educational campaigns are fundamental for a healthier food choice [140] and lifestyles [141], mainly for young people [142] to obtain critical skills [143] in making more informed decisions [144]. Educational campaigns to inform and create skills in consumers to deal with the abundance in daily advertisements are crucial in preventing health problems related to ill-informed consumption, mostly those related to obesity and diabetes. Another question concerns lifestyles that need to be adjusted in order to be healthier and prevent other diseases associated with an unbalanced diet. Cancers and cardiovascular diseases are examples of civilizational diseases related to population lifestyles and social contexts. The media could better support these healthier campaigns [145], considering its influence on adolescents [146], for example, in terms of food choices [147].

On the other hand, it is important to increase the social conscientiousness of the companies which support self-regulatory approaches. Public health policies may play an important role here to influence companies to voluntarily improve their social responsibility concerning the negative implications of marketing practices that promote the consumption of unhealthy foods [148]. Sugar and salt are among the main nefarious ingredients in unhealthy products [149], having several impacts on society’s dynamics, and they are sometimes presented on packaging along with other information in a misleading way [150]. The design of adjusted healthy food policies needs multidisciplinary approaches [151] that consider the several human dimensions [152], in which, of course, health professionals should be included [153]. Scientific research may also bring about significant insight and support here [154]. Children’s health, changing industry practices, intervention from public institutions, and consumer support are all consensual dimensions for the several stakeholders to promote healthier food production and choice [155].

Social condition has a great impact on food choices [156]. Indeed, the social and economic contexts have direct implications on the amount of income available to consume and on the level of prices afforded. However, in some cases, retailers are not clearly informed about the impacts of the price changes on their sales [157]. Food may also be used as an expression of social identity and a way to make a difference from the mainstream [158].

In general, food choice patterns followed by consumers are similar to those considered in other decisions of their lives [159]. In fact, consumers concerned with sustainability tend to consume foods of a higher quality and are less vulnerable to promotional advertisements [160]. The consumption patterns of these more sustainable consumers may be considered by, for example, policymakers as benchmarks and practices to be spread over other social segments. It is important to know the several dimensions related to food choices and consumption in order to promote more balanced lifestyles. For example, Chinese teenagers are influenced, in their food choices, by personal, family, peer, and retailer frameworks and the following features were highlighted as influencing their options: nutrition, safety, taste, image, price, convenience, and fun [161]. The social dimensions around the world are very different, and any adjusted approach needs to consider and be aware of the local particularities.

### 3.7. Brand and Branding

Brands and branding are fundamental instruments for an effective marketing plan in each step of the food chain [162]. From production to retailers’ markets, brands are crucial to create value added and to differentiate products from their competition. Only with brands is it possible to carry out a marketing strategy across all dimensions.

Commercial brands are more important for the brand-schematic consumers than for brand-aschematic consumers. The brand-aschematic consumers, in wine markets, for example, give greater importance to the Protected Designation of Origin label and the associated categories [163]. The wine market is a very complex context, due to its great number of individual and certified brands. Markets with a great diversity of brands may confuse consumers when they want to make a choice. In these cases, the main challenge is to have a brand that may be easily identified, amongst many others, and be positioned in the mind of the customers. Consumers, in general, maintain two brands by category in their minds, and the great task is to be included as one of these two brands. Here, positioning approaches are crucial for an efficient branding [164].

Credence features are decisive for the marketing of food, and the brand itself is among these characteristics jointly with organic foods, health, and ingredients [165]. The branding processes usually create ties of confidence and loyalty with consumers to maintain the market and the respective sales. These dimensions distinguish the concerns and objectives of sales technicians from marketing professionals. In addition, the scientific literature highlights that consumer satisfaction is interrelated with their behaviour and loyalty [166], showing that consumer loyalty is, indeed, a central dimension in marketing strategies and that brands are crucial in creating ties of confidence [167]. However, loyalty and satisfaction of consumers are, also, influenced by their lifestyle and personality [168].

Iconic and old brands, such as Coca-Cola, are examples of market drivers [169] and may bring important contributions for strategic plans to lead consumers towards a more adjusted and healthy consumption, principally among children and youths. On the other hand, the display of brand characters has an important impact on consumer choice, and this deserves special attention from the several stakeholders for healthier food consumption [170].

## 4. Discussion and Conclusions

The study presented here aimed to highlight the main contributions from the literature concerning the dimensions related to the interrelationships between food marketing and consumer choice. For this purpose, 147 documents from the Scopus database were considered in a search carried out on 16 October 2020 for the topics “food marketing” and “choices”. These documents were first analysed through bibliographic characterisation and after surveyed by literature review.

The bibliographic data reveals that there are opportunities to explore regarding these topics, considering the annual average number of documents published, the subject areas addressed, and the countries of the authors’ affiliation. On the other hand, there is great relatedness between food marketing and human obesity, especially in young people. In fact, the literature review highlighted that there is a great concern from several stakeholders about the impact of marketing strategies on the health of children and adolescents.

**Figure 6:**
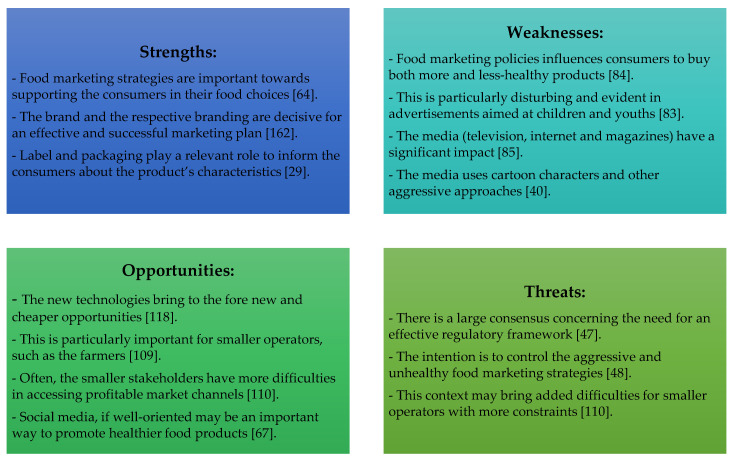
SWOT (strengths, weaknesses, opportunities, and threats) analysis to summarise the literature review.

The literature review may be summarised in a SWOT (strengths, weaknesses, opportunities, and threats) analysis approach, to better highlight the main insights, principally considering food marketing and consumer choice when building the matrix (see Figure 6).

Figure 6 shows that adjusted food image and name approaches, interrelated with the label, packaging, and brand, are crucial for a successful marketing strategy [6]. However, these powerful marketing instruments are often used by companies, through the media, to promote unhealthy food, especially for children and adolescents [49]. In parallel, new technologies and social media offer new and attractive opportunities for smaller operators, opening up new channels for them to communicate with consumers [118]. Nonetheless, these smaller stakeholders may be those most affected by restrictive policies to mitigate negative food marketing impacts on consumer health [47].

Traditionally, prices are amongst the most influential factors that condition consumption, including food choices, and the economic theory confirms this influence. Nonetheless, there are specific segments and new tendencies where quality, healthy attributes, and sustainability aspects are emergent dimensions. The sociodemographic, cultural, and behavioural domains also play their part in food consumption and preferences. This explains, in part, the emerging importance of neurosciences in marketing plans. In the universe of food marketing and consumer choice, it is important to highlight the relevance of the agricultural sector and its particularities, in the production of commodities, which condition the definition of effective marketing plans for the entire sector.

In terms of practical implications, it seems to be consensual that food marketing strategies have relevant implications on human health, and this framework deserves special attention from several stakeholders, particularly in the design of more adjusted policies in a standard way across countries, through World Trade Organization and World Health Organization negotiations. However, these regulations should be designed in order to have the right desired effect and avoid worsening the fragile context of smaller producers.

For future studies, it would be advisable to survey several stakeholders with regard to suggestions for designing new and efficient policies and regulations, so as to obtain a more adjusted regulatory framework and increase the operators’ compliance.
